# High steady-state column density of I(^2^P_3/2_) atoms from I_2_ photodissociation at 532 nm: Towards parity non-conservation measurements

**DOI:** 10.1038/srep33261

**Published:** 2016-09-15

**Authors:** G. E. Katsoprinakis, G. Chatzidrosos, J. A. Kypriotakis, E. Stratakis, T. P. Rakitzis

**Affiliations:** 1 Institute of Electronic Structure and Laser, Foundation for Research and Technology-Hellas, 71110 Heraklion-Crete, Greece; 2 Department of Physics, University of Crete, 71003 Heraklion-Crete, Greece

## Abstract

Steady-state column densities of 10^17^ cm^−2^ of I(^2^P_3/2_) atoms are produced from photodissociation of I_2_ vapour at 290.5 K using 5 W of 532 nm laser light. Recombination of the I(^2^P_3/2_) atoms at the cell walls is minimized by coating the cell surface with a hydrophobic silane (dimethyldichlorosilane/DMDCS). Operation at room temperature, and at an I_2_ vapour pressure of ~0.2 mbar, without using a buffer gas, allows relatively low Lorentz and Doppler widths of ~2*π* × 1.5 (FWHM) and ~2*π* × 150 (HW at 1/*e*^2^) Mrad/s, respectively, at the 
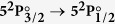
 M1 transition of atomic iodine at 1315 nm. These high column densities and low linewidths are favorable for parity nonconservation optical rotation measurements near this M1 transition. Furthermore, as the cell is completely sealed, this method of production of high-density ^127^I(^2^P_3/2_) atoms is also compatible with using iodine radioisotopes, such as for the production of high-density ^129^I(^2^P_3/2_).

The measurement of weak absorption or polarimetric signals, associated with forbidden transitions, requires large effective atomic or molecular column densities, typically of around ~10^15^ cm^−2^ or higher. High atomic densities are usually produced using high-temperature ovens (such as for most metals). For example, parity nonconserving optical rotation was measured in Tl, Bi, and Pb^1^,^2^,^3^,^4^, using column densities of ~10^19^ cm^−2^, produced by heating cells of length ~1 m to temperatures of about 1300 K (giving Doppler widths of ~2*π* × 250 Mrad/s). Discharge lamps are used to produce atomic radicals from diatomic dissociation, such as ~2 × 10^16^ cm^−2^ O(^3^P_*J*_) from discharge lamps using O_2_^5^, and ~10^16^ cm^−3^ I(^2^P_3/2_) atoms from I_2_^6^,^7^,^8^. However, both oven and discharge lamp methods usually require precursor and/or buffer gases, with pressures of around 20–50 mbar (giving Lorentz widths of about 2*π* × 20–200 Mrad/s).

PNC optical rotation has only been measured for Tl, Bi, and Pb, which all satisfy the following important criteria: strong and accessible M1 transitions (all happen to be near 1.28 *μ*m); large values for the ratio *R* = Im[*E*1_PNC_]/*M*1 (between 10^−7^–10^−6^); and can be produced at large column densities, *ρ*_*c*_ ~ 10^19^ cm^−2^. Together, these factors allowed the production of PNC optical rotation signals of ~1 *μ*rad, and their measurement with a precision of about 1%. The maximum PNC optical rotation angle *ϕ*_max_ is proportional to *R*; it is proportional to *ρ*_*c*_ for small optical depths 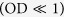, and proportional to (*ρ*_*c*_)^1/2^ for large optical depths 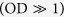
9; finally, *ϕ*_max_ generally increases with decreasing Lorentz and Doppler widths^9^. Therefore, *ϕ*_max_ can be increased by increasing *R* (i.e. by choosing a transition with large *R*) and *ρ*_*c*_ (i.e. by maximizing the density and pathlength), while decreasing the Lorentz width (e.g. decreasing pressure) and the Doppler width (e.g. decreasing temperature). Not all of these changes can be performed simultaneously, and an optimum combination of these experimental conditions must be found.

Recently, our group has proposed a method for the measurement of parity nonconserving (PNC) optical rotation using a novel bowtie cavity^9^,^10^,^11^ to enhance the effective column density by the number of cavity passes (~1000), while also introducing signal reversals which allow the isolation of the PNC signals from backgrounds. The operation of the cavity has been demonstrated through the measurement of chiral optical rotation of gases, liquids, and thin films^12^,^13^. This signal enhancement, and the background suppression and subtraction procedures, open the way for PNC optical rotation measurements in other atomic and molecular systems, which have smaller values of *R* and/or can be produced at smaller column densities, such as I(^2^P_3/2_)^10^ and metastable Hg and Xe^11^, molecular oxygen^14^, and other diatomic molecules^15^. For example, for the I(^2^P_3/2_) → I(^2^P_1/2_) M1 transition at 1315 nm, *R* = 0.8 × 10^−8^, which is about 20 times smaller than that of the Tl(^2^P_1/2_) → Tl(^2^P_3/2_) M1 transition at 1.28 *μ*m: *R* = 1.5 × 10^−7^. However, Katsoprinakis *et al*.^10^ proposed to compensate for this smaller value of *R* by using large cavity-enhanced pathlengths (~1000 m) to achieve column densities of about 10^20^ cm^−2^ and higher, but also by generating the I(^2^P_3/2_) atoms from the photodissociation of I_2_ molecules using 532 nm laser light, at low pressure and near room temperature, so that the Lorentz and Doppler widths can be significantly reduced, to ~2*π* × 1 Mrad/s and ~2*π* × 150 Mrad/s, respectively. In addition, the production of high-density atoms through photodissociation at room temperature is more convenient and more compatible with an optical cavity experiment than using a high-temperature oven.

The aim of this paper is to demonstrate the feasibility of producing I(^2^P_3/2_) atoms from I_2_ photodissociation, at high single-pass column densities of at least 10^17^ cm^−2^, while also achieving low Lorentz and Doppler widths. This method depends critically on minimizing the sticking and recombination of I(^2^P_3/2_) at the cell walls. We study the dependence of the produced atomic I(^2^P_3/2_) density on the photodissociating laser power, and investigate the effects of varying molecular iodine density and of various coatings of the iodine cell walls.

## Results

We consider the simplest possible model for the production of atomic iodine, assuming all iodine is either in the ground molecular state (denoted as I_2_) or in the ground atomic state (denoted as I). We assume that the population of excited states of I_2_ and production of I_3_ molecules are negligible (such assumptions have been used in previous studies to determine the rate constant *k*_*r*_ for the recombination of iodine atoms^16^,^17^). In this case, the rate equation governing the production rate of atomic iodine from photodissociating I_2_ is given by^10^,^17^:


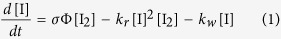


where [I] and [I_2_] are the atomic and molecular iodine densities, respectively, *σ* = 2.4 × 10^−18^ cm^2^ is the I_2_ photodissociation cross section at 532 nm^18^, Φ the green laser photon flux, related to the power of the green laser, *P*, through 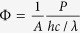, with *A* the cell cross sectional area, *k*_*r*_ is the gas phase three-body recombination rate, and *k*_*w*_ is the rate at which atomic iodine sticks to the cell walls, where it will eventually recombine into molecular iodine. We note that for the determination of Φ, the relevant area *A* is that of the cell, and not of the green laser beam (as the I and I_2_ diffusion times are smaller than the time it takes the photodissociating laser to deplete the molecular iodine). This is corroborated by the observation that the I-atom density is largely homogeneous (we vary the overlap of the IR and green beams by parallel translation of the IR in the radial direction of the cell), and insensitive to the size of the photodissociating green beam (maximal variations in the I-atom density of less than 20% are observed).

The first term on the right-hand side of Eq. (1) quantifies the photodissociation reaction, I_2_ + *hν* → I + I, where I ≡ I(^2^P_3/2_), and is the term which creates atomic iodine. It depends on the density of iodine molecules, on the flux of photons from the 532 nm laser source, and on the photodissociation cross section. The second and third terms are destruction terms. The second term describes the three-body recombination reaction I + I + I_2_ → 2I_2_, where two iodine atoms recombine in the presence of one iodine molecule. Other particles may act as third bodies in this process, but molecular iodine is by far the most effective^19^ (in particular, the recombination rate for the 3I → I_2_ + I is much smaller). The last term describes the sticking of iodine atoms on the container walls, i.e. I + wall → I_wall_. This process eventually leads to recombination of two iodine atoms into a molecule, i.e. I_wall_ + I_wall_ → I_2_, and is thus a source term for molecular iodine. The produced atomic iodine density depends on the photon flux of the photodissociating laser, Φ, the temperature, which has an impact predominantly on the molecular iodine density, [I_2_], but also on the *k*_*r*_ and *k*_*w*_ recombination parameters, and thus on the chemical properties of the cell surfaces which come in contact with I and I_2_, and which determine the recombination rate, *k*_*w*_.

The steady state solution of Eq. (1) is:


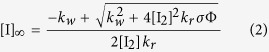


In the absence of the photodissociating green photon flux, Φ = 0, at a given temperature and for a specific cell, and after equilibrium has been reached, the molecular iodine density, [I_2_], is a constant determined by the vapour pressure of the solid iodine pellet inside the I_2_ holder area, and the temperature of the coldest spot the iodine vapour comes in contact with. We measure the absorption of a low-power blue led (center *λ* = 466 nm, FWHM = 24 nm), to determine the density of the molecular iodine vapor inside the cell. We use the Beer-Lambert law, 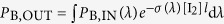, where *P*_B,IN(OUT)_ is the incident (transmitted) blue led power, *σ* is the absorption cross section of I_2_, and *l* = 123 cm is the length of the cell. The lineshape of *P*_B,IN_(*λ*) comes from our own measurements of the spectrum of the blue led using an Ocean Optics USB4000 spectrometer, while the wavelength dependence of *σ*(*λ*) is from^20^. From the absorption measurements, we get [I_2_] = (5.45 ± 1.9) × 10^15^ cm^−3^. Assuming literature values^21^, the ambient temperature corresponding to the measured density is found to be *T* = 290.5 ± 0.1 K.

We aim at determining the dependence of atomic iodine density on the power of the photodissociating green laser, on the molecular iodine density, and on the different chemical coatings of the inner surface of the cell. To this end we acquire transmission spectra of the IR laser as its frequency is scanned across the 
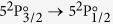
 transition of atomic iodine, for various powers of the photodissociating green laser, for various coatings of the inner surface of the iodine cell, and for molecular iodine vapour densities corresponding to two different temperatures, 290.5 K and 273 K (for the same cell). The experimental setup used is described in the Methods section, and is a typical pump-probe setup, with the CW photodissociating green laser (pump) and the IR laser (probe) counter-propagating and overlapping. The produced steady-state density of atomic iodine, as well as the characteristic widths of the two main broadening mechanisms, are obtained by fitting the acquired IR transmission spectra using the atomic absorption theory briefly presented in the Methods section.

### Absorption at Room temperature

**Figure 1:**
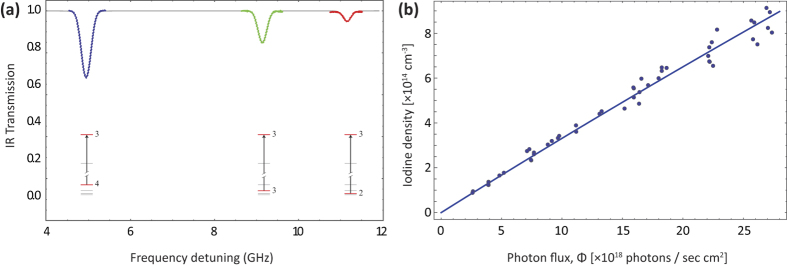
(**a**) Each *F* → *F*′ absorption peak is fitted independently, and an iodine density value is extracted. Colored, dotted features are experimental transmission data; the solid line is the fitted curve. (**b**) Atomic iodine density vs photodissociating laser photon flux, for a DMDCS coated cell at 290.5 K. Eq. (2) is used to fit the data (solid line).

We vary the green laser power up to its maximum value of 5 W and record the transmission spectra for the IR laser, as it is scanned across the 
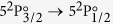
 transition of I (see Methods section). Each of the transmission peaks is fitted independently, as shown in Fig. 1a, to yield a value for the density of atomic iodine. The produced atomic iodine density is plotted in Fig. 1b versus the incident photodissociating laser photon flux, for a dimethyldichlorosilane (DMDCS) coated cell, at *T* = 290.5 K. The fitting of the data in Fig. 1b is performed using Eq. (2).

For the fit, we use the literature value for *k*_*r*_ at *T* = 290.5 K, *kr*|_290.5K_ = 4.4 × 10^−30^ ml^2^ s^−1 ^16, and the measured molecular iodine density to obtain the wall-sticking rate, *k*_*w*_ at 290.5 K:


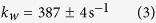


From Fig. 1b we notice that the [I] − Φ curve is mostly linear for high green laser power. From Eq. (2) we see that if the *k*_*r*_ term dominates, then the steady state atomic iodine density goes as the square root of the green photon flux, 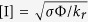, yielding a maximum atomic iodine density [I]_max_ ~ 3.9 × 10^15^ cm^−3^ for our maximum green laser power of 5 W. On the other hand, if the *k*_*w*_ term is dominant, the relation is linear, [I] = *σ*[I_2_]Φ/*k*_*w*_. The experimental curve for the DMDCS coating doesn’t show too visible a curvature, implying that the loss of atomic iodine occurs mostly as adsorption to the walls. For the rest of the coatings investigated, the wall adsorption was even more dominant as a loss mechanism.

For the DMDCS coated cell at 290.5 K, the steady state atomic iodine density achieved at peak green laser power, [I] = (0.89 ± 0.06) × 10^15^ cm^−3^, is only a factor of ~4 lower than the maximum value in the absence of wall recombination, [I]_max_, given above.

We note that a somewhat better fit to the data can be obtained by adding more detailed processes, such as the formation of an I_3_ intermediate^19^, adding a dependence to *k*_*w*_ on [I], or including the electronic excitation of I_2_ molecules from absorption of 532 nm light. However, the study of such effects is left for future work, particularly for measurements at higher [I] densities, where contributions that deviate from Eq. (2) should appear more strongly.

Finally, we note that, although it is known that angular momentum of I(^2^P_3/2_) atoms are aligned due to the photodissociation dynamics^22^, we observe no evidence of alignment (i.e. no difference in the absorption spectra is observed, for the photodissocation and probe laser polarization directions being parallel, compared to being perpendicular). This depolarization is caused by the thousands of collisions each atom suffers, with I_2_ molecules and the cell surface, before recombination.

### Effect of surface coatings and [I_2_] on produced I density

#### Coatings

**Figure 2:**
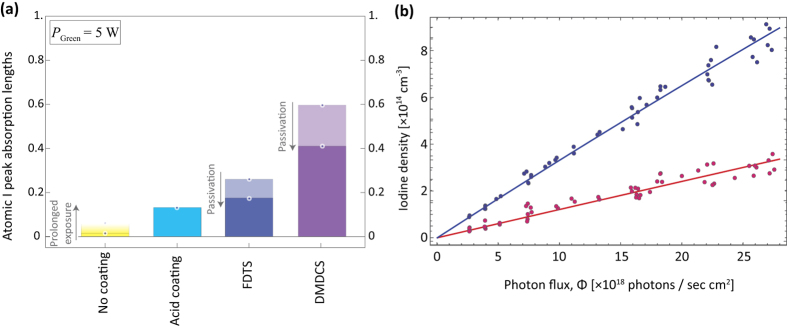
(**a**) Comparison of the peak absorptivities observed for various coatings of the inner glass cell walls. The DMDCS coating produced the best results, although passivation by molecular iodine seems to hinder its long-term performance. (**b**) Comparison of atomic iodine densities produced at 0 °C (red) and 290.5 K (blue). Lowering the I_2_ pellet temperature, and therefore lowering the I_2_ vapour pressure, leads to lower atomic iodine densities.

We measured I-atom density for five different cell surfaces: paraffin, dichlorodimethylsilane (DMDCS/Si(CH_3_)_2_Cl_2_), perfluorodecyltrichlorosilane (FDTS/C_10_H_4_Cl_3_F_17_Si), dilute phosphoric acid (H_3_PO_4_), and, of course, no coating. Paraffin coating has been widely used in atomic physics for many decades^23^, acid coating has been used to prevent iodine atom cell-wall recombination^17^,^24^, whereas DMDCS and FDTS coatings are used routinely on glass^25^. Absorption measurements were performed for the various coated cells, and the corresponding peak absorption values, which are directly proportional to the atomic iodine densities, are presented in the bar-chart of Fig. 2a. Although the performed study was far from exhaustive and long term performance (over months) of the coatings was not determined, from Fig. 2a we can draw some important conclusions:

- The best wall-recombination suppression was achieved with the DMDCS coating.
- FDTS, which was applied similarly, did not exhibit the same levels of performance. Moreover, both the DMDCS and the FDTS coatings started to deteriorate over a few days period, most probably due to passivation of the surface by molecular iodine.
- Our first try in applying a paraffin coating on the cell walls resulted into thick layers with rugged surfaces, which caused iodine to stick at very high rates. Performance was similar to no coating at all, if not worse. Applying into thiner and smoother layers might have resulted in better performance, but, since DMDCS provided an excellent combination of ease of application and wall-sticking-prevention operation, paraffin was not investigated further.
- Application of dilute phosphoric acid performed better than no coating, however it was not investigated further, especially as the application of the DMDCS was much easier in comparison.
- The uncoated cell exhibited the worst performance, yielding atomic iodine densities more than an order of magnitude smaller than the ones obtained in the DMDCS-coated cell. Continued exposure to molecular iodine, though, improved these values, possibly due to coating of the walls with solidified molecular iodine.

#### I_2_ density

For the DMDCS coated cell, we repeated the absorption measurement procedure with the I_2_ holder immersed in ice. The temperature of the created cold spot (0 °C) determines the vapour pressure of I_2_ inside the cell. However, this only affects the density of molecular iodine, and not the remaining parameters (cell temperature, recombination rates etc.), as the vapour is quickly thermalized. Varying the green laser power and repeating the fitting process for each individual hyperfine absorption peak, we arrive at the results plotted in Fig. 2b, where the *room* temperature results of Fig. 1b are plotted in the same figure for comparison. We see that reducing the temperature leads to the production of lower atomic iodine densities, due to the reduced molecular iodine vapour pressure. Therefore, raising the temperature is expected to increase the production of atomic iodine, until the photodissociating light is depleted.

## Conclusions

Our measurements demonstrate the steady-state production of I densities of about 10^15^ cm^−3^, and column densities of about 10^17^ cm^−2^, using 5 W of 532 nm light, and cells coated with DMDCS. Even higher I densities of about 10^16^ cm^−3^ should be possible by using higher 532 nm laser power (e.g. 50–200 W commercial lasers are available), and by improving the cell coatings further. Increased photodissociation will also reduce the Lorentz width of the 1315 nm transition, which is beneficial for polarimetry applications^10^: indeed, collisional broadening by I_2_ is about 2*π* × 7.7 (Mrad/s)/mbar, and by I about 2*π* × 3.2 (Mrad/s)/mbar^26^. At our current I_2_ pressure of ~0.2 mbar, the Lorentz linewidth is ~2*π* × 1.5 Mrad/s. Further photodissociation (e.g. decreasing I_2_ to less than 1 × 10^15^ cm^−3^, to produce [I] close to ~5 × 10^15^ cm^−3^) can reduce the linewidth to below 2*π* × 0.5 Mrad/s. This iodine-atom source can be used for various PNC measurements in iodine atoms, such as for the measurement of the weak nuclear charge, *Q*_weak_, nuclear spin-dependent PNC effects (needed to resolve the inconsistencies between such measurements in Cs and Tl^1^,^2^,^27^, and with theoretical calculations^28^,^29^,^30^,^31^), and for isotope-dependent PNC effects^32^,^33^. This method seems promising for the production of high atomic densities from the photodissociation of other diatomic molecules, such as from O_2_ or Br_2_.

## Methods

**Figure 3:**
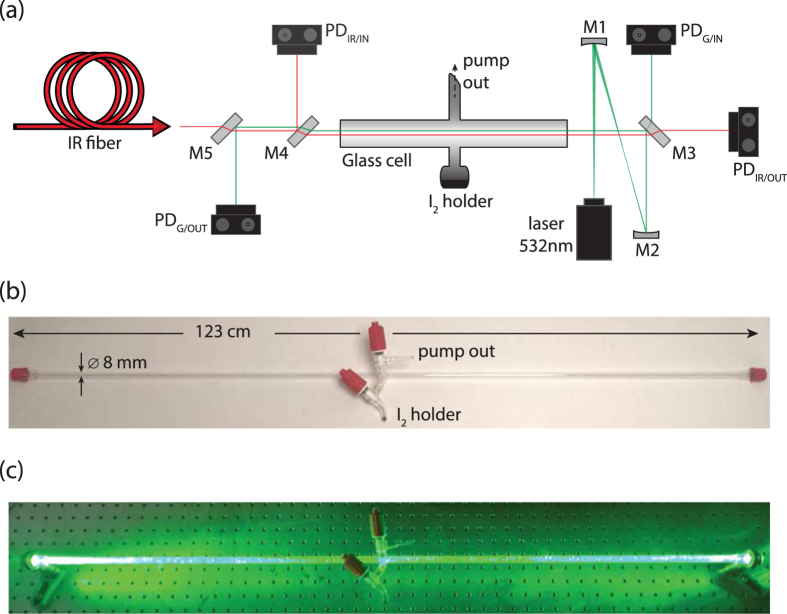
(**a**) Schematic of the experimental setup used for the study of atomic iodine production from photodissociation of molecular iodine at 532 nm. (**b**) The iodine glass cell. (**c**) The iodine cell with the green photodissociating laser passing through it.

**Figure 4:**
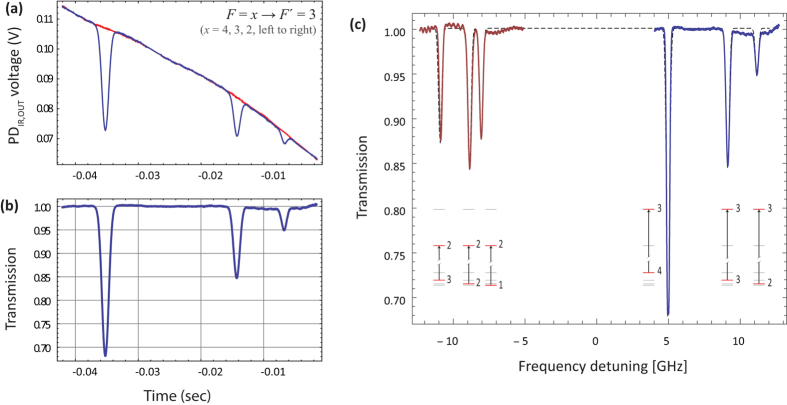
(**a**) Typical IR laser scan over the *F* → *F*′ = 3 group of the 
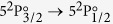
 transition, with the photodissociating green light ON (blue) and OFF (red/background measurement). (**b**) Resulting normalized absorption profile. (**c**) Solid lines: Combined *F* → *F*′ = 2 (left) and *F* → *F*′ = 3 (right) absorption measurements. Dashed line: The simulated transmission spectrum of Fig. 5b. The horizontal axis has been converted to frequency units for this figure. This is the highest atomic iodine density we have recorded, [I] ~ 0.89 × 10^15^ cm^−3^, using the full power of the photodissociating laser (*P* ~ 5 W), and the DMDCS coated cell.

The experimental setup used is shown in Fig. 3a. The probe IR laser (Toptica Photonics AG DL-Pro, *P* ~ 50 mW, continuously tunable around 1315 nm) is coupled to an optical fiber, and the fiber output beam propagates through the iodine cell. The beam of the photodissociating green laser (EKSMA Optics DPSS-532N-5000, *P* ~ 5 W CW, *λ* = 532 nm, bandwidth ~2 nm) is collimated to about twice the size of the IR beam using the spherical mirrors M1 and M2, and is injected to the iodine cell, counter-propagating to and overlapping with the IR beam. The input and output powers of the IR (green) laser are monitored by the PD_IR/IN_ (PD_G/IN_) and PD_IR/OUT_ (PD_G/OUT_) photodiodes (Thorlabs GmbH, models DET10N for the IR and DET36A for the green), respectively. The photodiode readings on the oscilloscope are calibrated to correspond to the actual powers of the two lasers incident to and transmitted from the iodine vapour, taking into account the transmission losses of the two windows. Mirrors M3, M4 and M5 are coated for 532 nm, and induce minimal losses to the power of the IR laser traversing them. Mirror M4 is used to dump most of the output green power, so as to minimize injection of green light into the IR fiber, and thus negative feedback to the IR laser, and to avoid saturating the PD_G/OUT_ photodiode. The cell, shown in Fig. 3b, was a home design; it was 123 cm long, with an inner diameter of 8 mm, and was made of BK7. In the middle of the cell were the pump outlet and the molecular iodine holder, both with individual controlling valves, while two uncoated windows seal both ends. The vacuum pump connected to the pump outlet was an Agilent Technologies TriScroll 300, a dry-scroll pump not requiring oil for operation. The DMDCS (Sigma-Aldrich > 98.5%) and FDTS (Sigma-Aldrich > 97%) coatings were applied in Hellmanex cleaned cells, using the substance in liquid form and allowing it to evaporate overnight inside the cell, in a controlled, clean environment, at ambient atmospheric pressure. Iodine in crystalline form was acquired commercially from Sigma-Aldrich (99.999% trace metal basis; ID: 229695). The experimental procedure is outlined below:

1. Initially the cell is pumped down to ~10^−2^ mbar and care is taken to ensure that, after pumping, the leak rate of the cell is insignificant within the time frame of the measurement (~1 min).
2. The pump valve is closed and the iodine holder valve is opened. The vapour pressure of molecular iodine reaches equilibrium (~0.22 mbar) within a few seconds, as confirmed by the transmitted green laser power reaching a steady state value.
3. The IR laser is switched on and scanned across the 
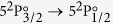
 atomic iodine transition. The basic atomic absorption theory relevant to this transition is given at the end of this section. The transmission spectrum is observed on the oscilloscope and is averaged for approximately 1 min.
4. The green laser is switched off, and a minute-long, time averaged measurement of the transmitted IR light is taken, in the absence of atomic iodine, as the laser is still scanned over the same frequency range. This is to be used as a 100%-transmission reference measurement.A typical measurement pair (green ON/OFF) is shown in Fig. 4a for the *F* → *F*′ = 3 group of the 
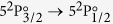
 transition, and the normalized absorption profile, resulting from the division of the green ON and OFF curves of (a), is shown in Fig. 4b. The combined measurements for the *F* → *F*′ = 2 and *F* → *F*′ = 3 transition groups is given in Fig. 4c, where the conversion of the horizontal axis from scan times to frequency detunings relative to the line center has been performed, using the known hyperfine constants of ^127^I^34^,^35^.
5. The cell is pumped down, the power of the green laser is varied, and the process is repeated.
6. The series of measurements described above is repeated for cells with different coatings and, for the same cell, with the iodine holder at two different temperatures.

A typical data set is obtained as follows: We vary the green laser power from zero up to its maximum value of ~5 W, and we extract the atomic iodine density from the optical depth calculated at each of the six observed absorption peaks (3 from the *F* → *F*′ = 2 and 3 from the *F* → *F*′ = 3 group). For each power value, we do this once as the IR laser is scanned from lower to higher frequencies, and once as it is scanned from higher to lower ones. We repeat the process as we decrease the green laser power from its maximum power down to zero. Note: from Fig. 1b we see that for relatively high green laser powers the measurement errors become significant. This was due to two main reasons: (a) instabilities of the IR power due to negative feedback of the green laser into the IR laser, and (b) variation of the IR laser power transmitted through the cell during the green-OFF measurements, as molecular iodine stuck to the windows forming an opaque layer (we note that with the green laser ON, the I_2_ layer at the windows would be removed via photodissociation). These two factors caused the ON and OFF curves of Fig. 4a to not coincide, hence introducing significant errors in the calculation of [I]. Despite this, we see that Eq. (2) describes the observed data well.

**Figure 5:**
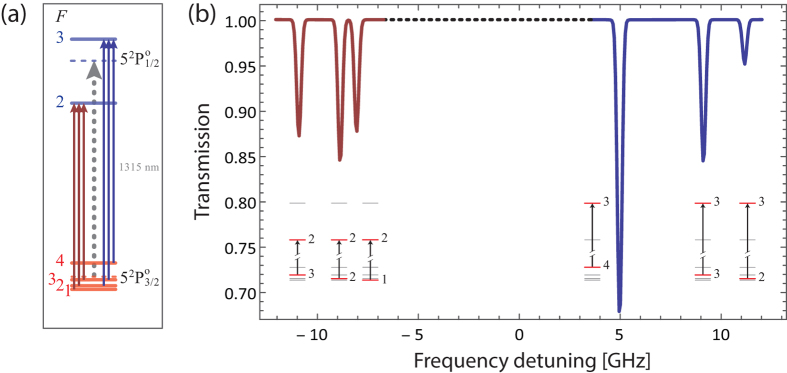
(**a**) Energy level structure of the 
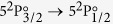
 M1 transition of atomic ^127^I. The two distinct hyperfine transition groups, *F* → *F*′ = 2 (left) and *F* → *F*′ = 3 (right), are indicated. The dashed lines mark the nominal transition frequency of 1315.27 nm, and the center-of-gravity energies of the ^2^P_3/2_ and ^2^P_1/2_ states. (**b**) Calculated transmission spectrum of an IR laser as its frequency is scanned across the resonance. For the calculation we assume [I] ~ 0.89 × 10^15^ cm^−3^, Γ = 2*π* × 3 Mrad/s, and Δ*ω*_D_ = 2*π* × 148 Mrad/s (half-width at 1/*e*^2^ value, for *T* = 290.5 K).

Finally, a short description of the atomic theory formalism used in this work is given here. The 
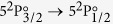
 transition of atomic iodine is a magnetic dipole transition with a nominal frequency of 1315.27 nm, between the ground 5^2^P_3/2_ and the metastable 5^2^P_1/2_ states, both of odd parity. The energy level breakdown of the transition is shown in Fig. 5a, and a typical transmission spectrum in Fig. 5b. There are two distinct groups of hyperfine transitions, the *F* → *F*′ = 2 (with *F* = 1, 2, 3) and *F* → *F*′ = 3 (with *F* = 2, 3, 4), red and blue detuned, respectively, by a few GHz from the nominal transition frequency.

For intensities well below saturation, the transmission of the IR light follows the Beer-Lambert law:


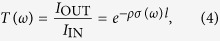


where *T* is the ratio of transmitted (*I*_OUT_) to incident (*I*_IN_) intensity, *ρ* the number density of the atomic iodine vapour, *σ* the absorption cross section, and *l* the length of the cell. All frequency dependence of the transmission is contained in the expression for the cross section, which is given by^9^:


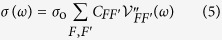


where *σ*_o_ is the *integrated absorption cross section*, *C*_*FF*′_ are geometrical factors, and 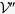 is the absorptive part of the Voigt profile:


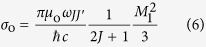



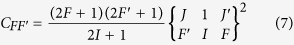



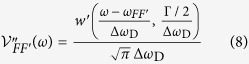


In the equations above, *ω*_*JJ*′_ is the nominal transition frequency and *ω*_*FF*′_ the frequency of the *F* → *F*′ transition, 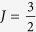, 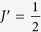 and 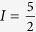, *M*_1_ ≡ 〈*M*_1_〉 ≡ 〈*J*||*μ*^(1)^||*J*′〉 = 1.15 *μ*_B_ is the reduced matrix element for the magnetic-dipole operator *μ*^(1)^^10^, *w*′ is the real part of the Faddeeva function, Γ is the FWHM homogeneous linewidth, and 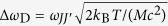 is the Doppler width.

## Additional Information

**How to cite this article**: Katsoprinakis, G. E. *et al*. High steady-state column density of I(^2^P_3/2_) atoms from I_2_ photodissociation at 532 nm: Towards parity non-conservation measurements. *Sci. Rep.* **6**, 33261; doi: 10.1038/srep33261 (2016).

## References

[b1] Vetter P. A., Meekhof D. M., Majumder P. K., Lamoreaux S. K. & Fortson E. N. “Precise Test of Electroweak Theory from a New Measurement of Parity Nonconservation in Atomic Thallium”. Phys. Rev. Lett. 74, 2658–2661, 10.1103/PhysRevLett.74.2658 (1995). 10057985

[b2] Edwards N. H., Phipp S. J., Baird P. E. G. & Nakayama S. “Precise Measurement of Parity Nonconserving Optical Rotation in Atomic Thallium”. Phys. Rev. Lett. 74, 2654–2657, 10.1103/PhysRevLett.74.2654 (1995). 10057984

[b3] Macpherson M. J. D., Zetie K. P., Warrington R. B., Stacey D. N. & Hoare J. P. “Precise measurement of parity nonconserving optical rotation at 876 nm in atomic bismuth”. Phys. Rev. Lett. 67, 2784–2787, 10.1103/PhysRevLett.67.2784 (1991). 10044554

[b4] Meekhof D. M., Vetter P., Majumder P. K., Lamoreaux S. K. & Fortson E. N. “High-precision measurement of parity nonconserving optical rotation in atomic lead”. Phys. Rev. Lett. 71, 3442–3445, 10.1103/PhysRevLett.71.3442 (1993). 10054978

[b5] Gupta M., Owano T., Baer D. & O’ Keefe A. “Quantitative determination of the *O*(^3^P) density via visible cavity-enhanced spectroscopy”. Appl. Phys. Lett. 89, 241503, 10.1063/1.2408655 (2006).

[b6] Azyazov V. N., Mikheyev P. A., Vorobyov M. V. & Ufimtsev N. I. “Properties of a DC glow discharge iodine atom generator”. Proc. SPIE 7131, 71310A, 10.1117/12.816461 (2008).

[b7] Mikheyev P. A., Shepelenko A. A., Voronov A. I. & Kupryaev N. V. “Atomic iodine production in a gas flow by decomposing methyl iodide in a dc glow discharge”. Quantum Electron 32, 1–4, 10.1070/QE2002v032n01ABEH002115 (2002).

[b8] Mikheyev P. A., Shepelenko A. A., Voronov A. I. & Kupryaev N. V. “Production of iodine atoms by dissociating CH_3_I and HI in a dc glow discharge in the flow of argon”. J. Phys. D 37, 3202–3206, 10.1088/0022-3727/37/22/024 (2004).

[b9] Bougas L., Katsoprinakis G. E., von Klitzing W. & Rakitzis T. P. “Fundamentals of cavity-enhanced polarimetry for parity-nonconserving optical rotation measurements: Application to Xe, Hg, and I”. Phys. Rev. A 89, 052127, 10.1103/PhysRevA.89.052127 (2014).

[b10] Katsoprinakis G. E., Bougas L., Rakitzis T. P., Dzuba V. & Flambaum V. V. “Calculation of parity-nonconserving optical rotation in iodine at 1315 nm”. Phys. Rev. A 87, 040101(R), 10.1103/PhysRevA.87.040101 (2013).

[b11] Bougas L., Katsoprinakis G. E., von Klitzing W., Sapirstein J. & Rakitzis T. P. “Cavity-Enhanced Parity-Nonconserving Optical Rotation in Metastable Xe and Hg”. Phys. Rev. Lett. 108, 210801, 10.1103/PhysRevLett.108.210801 (2012). 23003234

[b12] Sofikitis D., Bougas L., Katsoprinakis G. E., Spiliotis A. K., Loppinet B. & Rakitzis T. P. “Evanescent-wave and ambient chiral sensing by signal-reversing cavity ringdown polarimetry”. Nature 514, 76–79, 10.1038/nature13680 (2014). 25209661

[b13] Bougas L., Sofikitis D., Katsoprinakis G. E., Spiliotis A. K., Tzallas P., Loppinet B. & Rakitzis T. P. “Chiral cavity ring down polarimetry: Chirality and magnetometry measurements using signal reversals”. J. Chem. Phys 143, 104202, 10.1063/1.4930109 (2015). 26374026

[b14] Labzovsii L. N. “Effects of parity nonconservation in electronic spectra of molecules”. Sov. Phys. JETP 46, 853–858 (1977).

[b15] Sushkov O. P. & Flambaum V. V. “Parity breaking effects in diatomic molecules”. Sov. Phys. JETP 48, 608–611 (1978).

[b16] Ip J. K. K. & Burns G. “Recombination of Iodine Atoms by Flash Photolysis over a Wide Temperature Range. II I_2_ in He, Ar, Xe, N_2_, CO”. J. Chem. Phys. 56, 3155–3161, 10.1063/1.1677654 (1972).

[b17] Brewer L. & Tellinghuisen J. “Detection of Iodine Atoms by an Atomic Fluorescence Technique: Application to Study of Diffusion and Wall Recombination”. J. Chem. Phys. 54, 5133–5138, 10.1063/1.1674807 (1971).

[b18] Tellinghuisen J. “Least-squares analysis of overlapped bound-free absorption spectra and predissociation data in diatomics: The *C*(^1^Π_*u*_) state of I_2_”. J. Chem. Phys. 135, 054301, 10.1063/1.3616039 (2011). 21823694

[b19] Tellinghuisen J. & Phillips L. F. “Kinetics of I_2_ following photolysis at 1930 Å: temperature dependence of A’-state quenching”. J. Phys. Chem. 90, 5108–5120, 10.1021/j100412a046 (1986).

[b20] Saiz-Lopez A., Saunders R. W., Joseph D. M., Ashworth S. H. & Plane J. M. C. “Absolute absorption cross-section and photolysis rate of I_2_”. Atmos. Chem. Phys. 4, 1443–1450, 10.5194/acp-4-1443-2004 (2004).

[b21] Baxter G. P. & Grose M. R. “The vapor pressure of iodine between 50° and 95°”. J. Am. Chem. Soc. 37, 1061–1072, 10.1021/ja02170a007 (1915).

[b22] Chestakov D. A., Parker D. H., Vidma K. V. & Rakitzis T. P. “Photofragment alignment in the photodissociation of I_2_ from 450 to 510 nm”. J. Chem. Phys. 124, 024315, 10.1063/1.2147203 (2006). 16422591

[b23] Bouchiat M. A. & Brossel J. “Relaxation of Optically Pumped Rb Atoms on Paraffin-Coated Walls”. J. Phys. Rev. 147, 41–54, 10.1103/PhysRev.147.41 (1966).

[b24] WassermanB. E., FalconerW. E. & YagerW. A. “Direct Predissociation of I_2_ 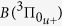 ”. J. Chem. Phys. 49, 1971–1972, 10.1063/1.1670340 (1968).

[b25] Wasserman S. R., Tao Y.-T. & Whitesides G. M. “Structure and reactivity of alkylsiloxane monolayers formed by reaction of alkyltrichlorosilanes on silicon substrates”. Langmuir 5, 1074–1087, 10.1021/la00088a035 (1989).

[b26] Engleman R. Jr., Palmer B. A. & Davis S. J. “Transition probability and collision broadening of the 1.3-*μ*m transition of atomic iodine”. J. Opt. Soc. Am. 73, 1585–1589, 10.1364/JOSA.73.001585 (1983).

[b27] Wood C. S., Bennett S. C., Cho D., Masterson B. P., Roberts J. L., Tanner C. E. & Wieman C. E. “Measurement of Parity Nonconservation and an Anapole Moment in Cesium”. Science 275, 1759–1763, 10.1126/science.275.5307.1759 (1997). 9065393

[b28] Haxton W. C. & Wieman C. E. “Atomic Parity Nonconservation and Nuclear Anapole Moments”. Annual Rev. Nucl. Part. Sci. 51, 261–293, 10.1146/annurev.nucl.51.101701.132458 (2001).

[b29] Ginges J. S. M. & Flambaum V. V. “Violations of fundamental symmetries in atoms and tests of unification theories of elementary particles”. Phys. Rep. 397, 63–154, 10.1016/j.physrep.2004.03.005 (2004).

[b30] Porsev S. G., Beloy K. & Derevianko A. “Precision Determination of Electroweak Coupling from Atomic Parity Violation and Implications for Particle Physics”. Phys. Rev. Lett. 102, 181601–181604, 10.1103/PhysRevLett.102.181601 (2009). 19518856

[b31] Porsev S. G., Beloy K. & Derevianko A. “Precision determination of weak charge of ^133^Cs from atomic parity violation”. Phys. Rev. D 82, 036008–036016, 10.1103/PhysRevD.82.036008 (2009). 19518856

[b32] Dzuba V. A., Flambaum V. V. & Khriplovich I. B. “Enhancement of P- and T-nonconserving effects in rare-earth atoms”. Z. Phys. D 1, 243–245, 10.1007/BF01436678 (1986).

[b33] Fortson E. N., Pang Y. & Wilets L. “Nuclear-structure effects in atomic parity nonconservation”. Phys. Rev. Lett. 65, 2857–2860, 10.1103/PhysRevLett.65.2857 (1991). 10042716

[b34] Drühl K. “Cross section and hyperfine structure of the atomic iodine (^2^P_1/2_ - ^2^P_3/2_) Raman transition”. Phys. Rev. A 26, 863–868, 10.1103/PhysRevA.26.863 (1982).

[b35] Payne D. S., Wilson G. J. & Devonshire R. “Non-thermal nuclear hyperfine populations in the products of a photodissociation reaction”. Chem. Phys. Lett. 332, 58–64, 10.1016/S0009-2614(00)01258-6 (2000).

